# eG Coated Stents Exhibit Enhanced Endothelial Wound Healing Characteristics

**DOI:** 10.1007/s13239-021-00542-x

**Published:** 2021-05-18

**Authors:** Belen Rodriguez-Garcia, Christophe Bureau, Abdul I. Barakat

**Affiliations:** 1 Hydrodynamics Laboratory, CNRS UMR7646, Ecole Polytechnique, Institut Polytechnique de Paris, Palaiseau, France; 2 AlchiMedics S.A.S, Paris, France

**Keywords:** Stent, Simulated arterial model, Wound healing, Automated image analysis, BuMA, Polymer brush coating

## Abstract

**Purpose:**

Despite their widespread use, a significant fraction of coronary stents suffer from in-stent restenosis and stent thrombosis. Stent deployment induces extensive injury to the vascular endothelium. Rapid endothelial wound closure is essential for the success of a stenting procedure. A recent study has demonstrated that the BuMA Supreme® sirolimus-eluting stent exhibits particularly attractive strut coverage characteristics. A unique feature of this stent is the presence of a thin brush layer of poly-butyl methacrylate (PBMA), covalently bonded to the stent’s cobalt-chromium frame *via* electro-grafting (*e*G™). The present study aimed to determine whether the PBMA coating has an effect on endothelial cell wound healing and stent strut coverage.

**Methods:**

We used an *in vitro* coronary artery model whose wall consisted of an annular collagen hydrogel and whose luminal surface was lined with a monolayer of endothelial cells. Mechanical wounding of the endothelial lining was preformed prior to deployment of a bare cobalt-chromium stent either with or without the PBMA layer. The migration of fluorescently labeled endothelial cells was monitored automatically over a period of 48 h to determine endothelial wound healing rates.

**Results:**

Quantitative assessment of endothelial wound healing rates within the simulated arterial model is achievable using automated image analysis. Wound healing is significantly faster (44% faster at 48 h) for stents with the PBMA eG Coating™ compared to bare metal stents.

**Conclusion:**

The PBMA eG Coating™ has the effect of promoting endothelial wound healing. Future studies will focus on elucidating the mechanistic basis of this observation.

**Supplementary Information:**

The online version contains supplementary material available at 10.1007/s13239-021-00542-x.

## Introduction

Millions of coronary stents are deployed in patients worldwide every year. However, despite this widespread use and significant progress over the past couple of decades, coronary stenting procedures continue to suffer from complications, most notably in-stent restenosis and stent thrombosis, that pose significant risk to patients. For instance, stent thrombosis risk has been estimated at ~ 1% after 1 year and approximately 0.2–0.4% per year thereafter, while rates of in-stent restenosis appear to be in the 5–10% range.^10^ There is evidence that stent-related complications depend on a complex combination of several considerations including stent materials, strut thickness, and detailed stent design^17^–^19^; however, the precise connection among these physical parameters and the incidence of complications remains incompletely understood.

Stent deployment induces significant injury to the vascular wall at the site of implantation including denudation of the vascular endothelium.^7^,^12^ Considering the role that normal, functioning endothelial cells (ECs) play in regulating smooth muscle cell (SMC) proliferation and in preventing thrombus formation, stent-induced endothelial damage is thought to be a significant contributor to stent-related complications. Thus, rapid endothelial wound healing is considered to be a critical factor in the success of a stenting procedure.

Endothelial wound healing post injury is enabled by a sequence of coordinated cellular events involving cell spreading, directional migration, and proliferation.^2^ Endothelial wound healing rates depend on a number of factors including the biochemical and biophysical characteristics of the substrate on which the cells migrate^11^,^21^ as well as the fluid mechanical environment to which the cells are subjected due to blood flow.^1^,^16^,^20^ Therefore, the observed differences in clinical performance among stents that possess different designs, strut thicknesses, and surface material properties may stem from differences in endothelial wound healing rates. Establishing the effect of stent characteristics on endothelial wound healing and stent strut coverage is thus of interest. Two particularly relevant recent studies in this regard used optical coherence tomography (OCT) measurements in a randomized trial to demonstrate that the BuMA Supreme® sirolimus-eluting stent (SES) (SINOMED, Tianjin, China) exhibits superior stent coverage in comparison to other widely-used drug-eluting stents such as the Xience V stent (Abbott Vascular).^6^,^23^ In human post-mortem histological analysis, strut coverage has been shown to be an independent predictor for late (> 30 days) stent thrombosis.^15^

**Figure 1:**
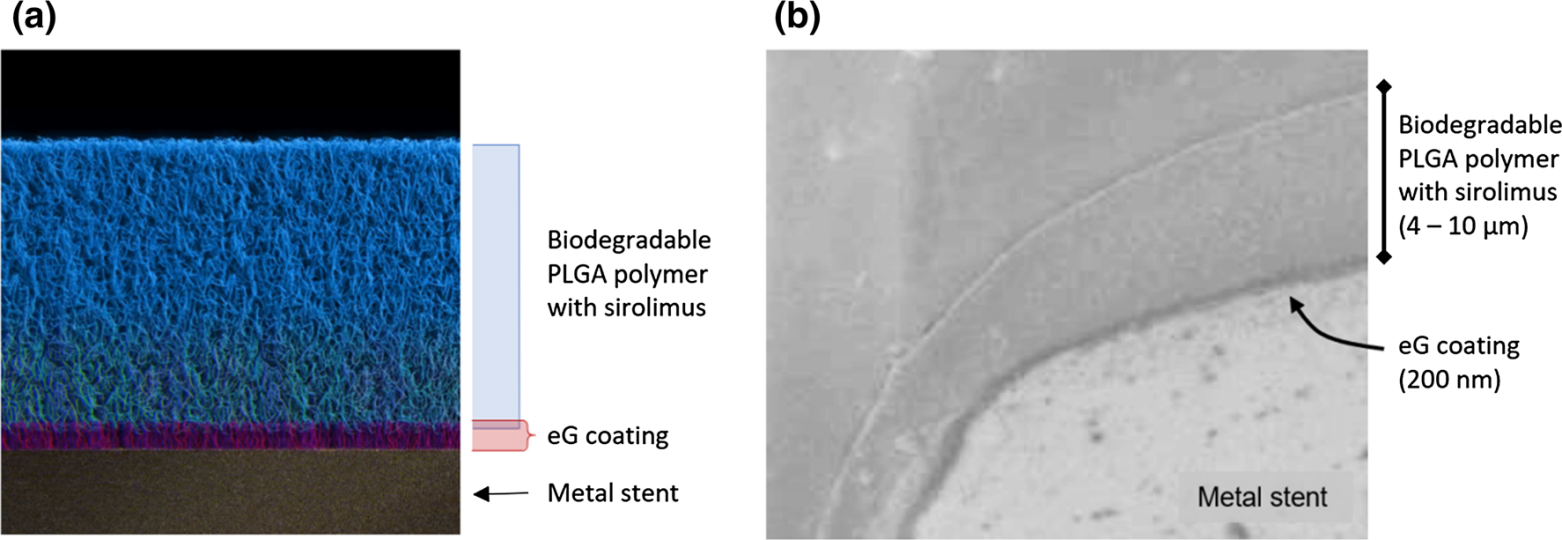
(a) Schematic of a cross-sectional representation of PBMA, covalently bonded to the Co–Cr surface *via* electro-grafting (*e*G™), and therapeutic coating of PLGA and sirolimus interdigitated into the eG Coating™. (b) SEM image of a BuMA Supreme SES strut cross-section, showing the 200 nm electo-grafted layer and 4–10 *µ*m therapeutic coating.

As depicted in Fig. 1, the BuMA Supreme® SES is made of a thin-strut (80 *μ*m) cobalt-chromium (Co–Cr) platform with a ~ 200 nm-thick eG Coating™: this coating is a brush layer of poly-butyl methacrylate (PBMA), covalently bonded to the Co–Cr surface *via* electro-grafting (*e*G™),^8^ top-coated with a 4–10 *μ*m-thick biodegradable coating of poly lactic-co-glycolic acid (PLGA) that releases sirolimus.^9^ One peculiarity of BuMA Supreme® is that it releases ~ 90% of its sirolimus payload in 28 days, which is faster than most existing SES. The improved strut coverage outcome of BuMA Supreme® has been attributed to this short release and to the fact that there is no polymer left behind, since the matrix hosting the drug is fully biodegraded.^25^ Asano *et al.* noted, however, that the superior performance of BuMA Supreme® vs. Xience V in terms of stent coverage extends beyond 1 month, i.e. after the drug release is complete. One possible explanation is that sirolimus is a cytostatic drug which inhibits the proliferation of ECs more effectively than SMCs^22^; thus, the sooner the drug disappears, the sooner ECs are able to proliferate and migrate and to thus promote strut coverage. This, however, would also hold true for SMCs and may account for the slightly higher late lumen loss observed with BuMA Supreme® in comparison to Xience V, even though restenosis rates remain low.

Another fundamental difference between BuMA Supreme® and other DES is the presence of the electro-grafted PBMA layer (eG Coating™). In the present study, we wished to explore if this base layer may play a role in promoting EC migration compared to a bare metal surface, thereby contributing to accelerated strut coverage during and after drug release. To this end, we used an *in vitro* coronary simulated arterial model that we had previously described and within which stents can be deployed and endothelial wound healing monitored in real-time.^3^ Briefly, this system consists of an annular collagen I hydrogel lined with a monolayer of ECs on its luminal surface. Steady or pulsatile flow matching human coronary flow parameters can be produced in the simulated arterial model and sustained for several days to weeks. A key distinguishing feature of this simulated arterial model relative to other engineered constructs is that it provides the ability to deploy stents. Fully compatible with both wide-field and confocal microscopy, the *in vitro* simulated arterial model enables live imaging of cells and dynamic monitoring of endothelial wound healing following stent-induced injury. Here, we use this simulated arterial model in combination with automated image analysis to directly compare EC migration and wound healing rates on a stent with the same design as the BuMA Supreme®, both with and without an electro-grafted layer in order to identify a possible interplay of the eG layer on the dynamics of strut coverage.

## Materials and Methods

### Cell Culture

Bovine aortic ECs (BAECs; gift of Dr. C. Boulanger, Georges Pompidou Hospital, Paris, France) were cultured at 37 °C and 5% CO_2_ in Dulbecco’s modified Eagle’s medium (DMEM; Invitrogen) supplemented with 10% fetal bovine serum (Invitrogen) and 1% penicillin/streptomycin (Invitrogen). Cells in passages 5–8 were used. Immediately prior to each experiment, ECs were labeled with red fluorescent Vybrant CM-DiI (Life Technologies) to facilitate time-lapse imaging.

### Collagen Hydrogel

Type I collagen was isolated from rat tail tendons as previously described.^3^ Briefly, tendons were manually extracted from tails, dissolved in 0.01 M hydrochloric acid (HCl; Sigma), and centrifuged at 30,000×*g* and 4 °C for 1 h. The supernatant was then cooled to – 80 °C, freeze dried, and stored at – 20 °C. Prior to experiments, lyophilized collagen was reconstituted to 12 mg/mL in 0.01 M HCl and stored at 4 °C. In order to mimic the arterial wall, a collagen hydrogel was prepared prior to each experiment by combining 1 mL cold acidic collagen solution with 1 mL cold neutralizing buffer containing 200 mL 10× concentrated DMEM (Sigma), 756 mL EC culture medium and 43.5 mL of 1 M sodium hydroxide (Sigma) for a final pH of 8.4, collagen concentration of 6.0 mg/mL.

### Simulated Arterial Model Design and Assembly

**Figure 2:**
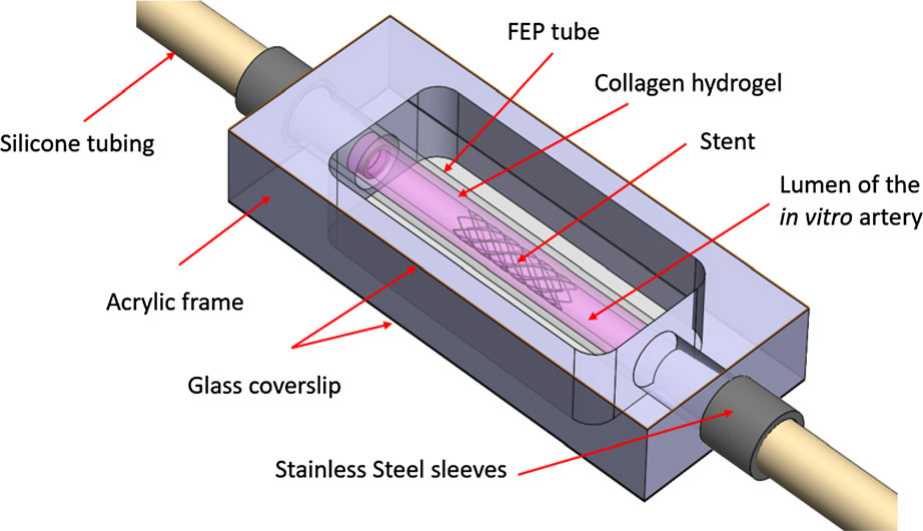
Schematic representation of the *in vitro* simulated coronary artery model.

The *in vitro* coronary simulated arterial model has been described in detail elsewhere.^3^ Briefly, fluorinated ethylene propylene (FEP) tubing (inner diameter 4.8 mm, wall thickness 0.8 mm, length 60 mm) (Fisher) was plasma treated for 45 s to activate hydroxyl groups on the surface, cross-linked with 1% polyethylenimine (Sigma) for 10 min to improve collagen hydrogel adhesion, and finally treated with 0.1% glutaraldehyde (Sigma) for 20 min to render the surface hydrophilic. The FEP was then thoroughly dried by aspiration and fitted at its ends with custom-machined stainless steel sleeves with concentric 3.0 mm ports. The neutralized collagen solution was pipetted into the FEP tubing which was fitted with a 3.0 mm-diameter stainless steel pin. The collagen solution was allowed to polymerize at 37 °C for 20 min, resulting in a hydrogel with a compression modulus of 4600 ± 1500 Pa and average pore diameter of 1.4 ± 0.1 *μ*m (mean ± SEM).^4^ After polymerization, the pin was removed, leaving a 3.0 mm-diameter channel centered in the hydrogel. Finally, the channel was seeded with a 1.8 × 10^6^ cells/mL suspension of BAECs. The seeding was performed in multiple stages over a period of 1 h, rotating the device between each stage. The cells formed a continuous monolayer covering the channel lumen within 24 h. The simulated arterial model was encased in an acrylic frame that provided support and facilitated live fluorescence imaging and quantification of biological response. The assembled device is shown schematically in Fig. 2.

In preparation for imaging, a 24 × 60 mm glass coverslip was glued to the bottom of the acrylic frame using vacuum grease. The device was filled with phosphate buffered saline (PBS) and sealed with a matching acrylic coverslip on the top of the frame to prevent evaporation of the PBS bath during the experiment. Because the refractive index of FEP closely matches those of PBS and cell-culture medium, this configuration enables acquisition of undistorted images using an epifluorescence microscope.

### Perfusion

After assembly, the *in vitro* simulated arterial model was subjected to preconditioning flow in an incubator (37 °C, 5% CO_2_) using cell culture medium. An initial flow rate of 0.016 mL/min, corresponding to a wall shear stress (WSS) less than 0.1 mPa (0.001 dyn/cm^2^), was applied. This flow rate was a compromise between having sufficiently high flow to replenish cell culture medium (necessary for maintaining cell viability) and sufficiently low flow to avoid cell detachment during endothelial adhesion and spreading. Over a period of 72 h, the flow rate was increased over three orders of magnitude in a stepwise fashion to ultimately achieve coronary-level flow without damaging the newly formed endothelium. After flow preconditioning, the *in vitro* arterial model was removed from the incubator, transferred to a recirculating flow loop and mounted on an epifluorescence microscope for imaging. As detailed in our previous work,^3^ the final flow rate applied to the *in vitro* artery, 40 mL/min, was designed for hydrodynamic similarity with the flow of blood in the human coronary artery. A time-averaged Reynolds number of 360 and a Womersley number of 8 with a pressure amplitude of 20 mmHg (2.7 kPa) were imposed. *In vivo,* both Reynolds and Womersley numbers vary with position in the coronary tree, but the values used here fall within the physiological range.^5^,^19^,^24^,^25^ The fluid properties used in computing the non-dimensional numbers were the density and dynamic viscosity of cell culture medium at 37 °C (1000 kg/m^3^ and 0.78 mPa s, respectively). The Reynolds number was computed based on the nominal channel diameter (3.0 mm), while the Womersley number was based on the channel radius (1.5 mm) and the nominal pump roller frequency (3.5 Hz). The mean shear stress on the endothelium is estimated to be 0.2 Pa (2 dyn/cm^2^). This estimate is based on the assumption of Poiseuille flow where the wall shear stress *τ*_w_ is given as: *τ*_w_ = 4 *µ*Q/πR^3^, where µ is the dynamic viscosity of the fluid (0.78 mPa s), *Q* is the volumetric flow rate (40 mL/min), and *R* is the channel radius (1.5 mm). The system was subjected to this flow condition for 24 h before stent deployment.

### Endothelial Wounding

*In vivo,* balloon angioplasty and stent deployment induce injury to the arterial wall and extensive EC denudation. The mechanisms by which balloon-induced EC injury occurs and its dependence on wall mechanical properties are not completely understood and are currently under study in our group. Results to date have revealed that soft substrates such as the collagen hydrogel used here can absorb a significant fraction of the compressive and shear stresses generated by balloon inflation and stent expansion, resulting in less endothelial denudation than in the case of stiffer substrates such as polydimethylsiloxane (PDMS) or a true arterial wall. Consequently, stents deployed in a simulated arterial model with a wall consisting of a collagen hydrogel do not damage the endothelium as extensively as the *in vivo* situation. To overcome this limitation, a thin metallic wire with a circular hook at its end was used to scratch a wound into the endothelium prior to stent deployment in order to simulate the wounding produced by the deployment of the stent in a real artery. It is recognized, of course, that the damage inflicted by this object is not identical to that induced by a stent; however, there are some important similarities between the two scenarios. *In vivo,* a stent damages the endothelium *via* a combination of mechanical scratching (shear stresses) due to stent and balloon sliding and normal stresses due to balloon inflation and stent expansion. The wire hook used in this study damages the endothelium *via* mechanical scratching; this is thought to be similar to the shear stresses generated by sliding stents and balloons. Stents were deployed within the simulated arterial model immediately after endothelial wounding.

### Stent Types Used and Deployment Procedure

Two types of stents were used: (1) a 15 mm-long bare (not drug-coated) Co–Cr stent having the same design and strut thickness (80 *µ*m) as the BuMA Supreme® SES and a nominal expanded diameter of 3 mm, henceforth referred to as “Bare BuMA” and (2) the same stent but with the 200 nm-thick PBMA layer electro-grafted onto its surface, termed “eG BuMA”.

**Figure 3:**
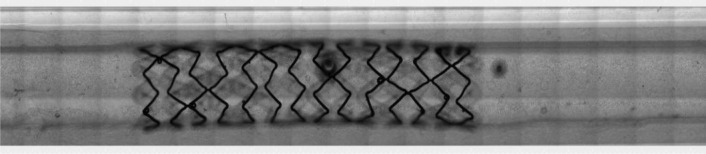
Phase contrast image of a stent implanted in the simulated arterial model. Note the slight deformation of the collagen after balloon inflation and stent deployment (due to stent over-expansion).

To deploy each stent within the simulated arterial model, the balloon catheter on which the stent was mounted was connected to an inflation syringe (Merit Medical), filled with PBS, purged of air and connected to a three-way valve in the flow loop. Flow in the *in vitro* simulated arterial model was momentarily halted, and the catheter was inserted through the three-way valve to the center of the *in vitro* artery and inflated to a pressure of 15 atm, over-expanding the stent by 10% to a diameter of 3.3 mm. Implantation data are shown in Table 1. This overexpansion was targeted in order to ensure stent apposition and retention within the artery during and after removal of the balloon. After deflation, the balloon catheter was withdrawn, and flow was restarted. Correct positioning of the stent and its uniform apposition were verified in brightfield (Fig. 3). This process can be visualized in the electronic supplementary material, video S1.

**Table 1:** Implantation data of the stent in the simulated arterial model.

Implantation data	
Pressure balloon inflation (nominal diameter)	8 atm
Overexpansion to stent anchorage	10%
Pressure to overexpansion	15 atm
Duration of balloon inflation	5 s
Nominal diameter pre-stent deployment	3 mm
Diameter after overexpansion	3.3 mm

### Time-Lapse Imaging

EC migration was monitored using time-lapse imaging on an epifluorescence microscope (Ti-E, Nikon) with a motorized stage, 1280 × 1024 CCD camera (Flash 4, Hamamatsu,) and ×4 magnification (1.66 *µ*m/pix). Images were acquired at 2 frames per hour after stenting. A halogen lamp was used for phase contrast illumination, while a mercury lamp was used for fluorescence illumination, with a TRITC filter for imaging the Vybrant CM-DiI-labeled ECs. At each time point, phase contrast and fluorescence images were acquired at each position of a 20 × 3 grid with 5% overlap in both *x*-*y* parameters, covering the entire *in vitro* simulated arterial model. Each image sequence was acquired with the focal plane at a plane tangent to the lumen. Image acquisition was controlled through µManager, an IMAGE-J plugin.^14^

### Statistical Analysis

Five independent experiments were performed for each stent type. In order to statistically compare endothelial wound healing rates between the two types of stents studied, an unpaired *t* test was performed in GraphPad at each measurement time point. *P* values < 0.05 were considered to be statistically significant.

## Results

### Automated Image Analysis Protocol for Quantifying Endothelial Wound Healing Rates

**Figure 4:**
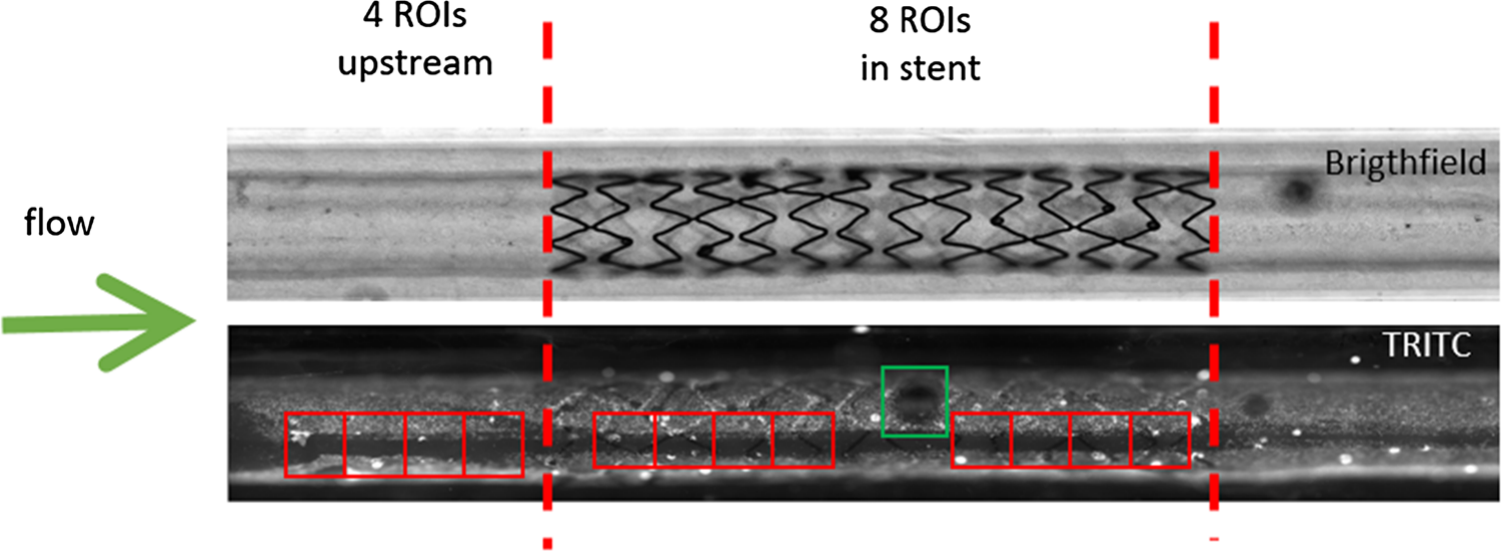
ROI selection in the *in vitro* simulated arterial model for endothelial wound healing quantification. Four ROIs were selected upstream of the stent, and 8 ROIs were defined in the stented region. ROIs were maintained over time to track the wound healing in the same regions. The green arrow denotes the direction of flow. The green box is an example of an area of collagen imperfection (air bubble in this case) which was not included in the analysis.

To quantify the time evolution of the area of the wound and thus to calculate the wound healing rate over time, we developed an automated image analysis protocol. In each experiment, 12 regions of interest (ROIs) were selected from the TRITC (endothelial) channel, each containing a well-defined section of the wound: four ROIs upstream of the stent, and eight ROIs within the stented zone (Fig. 4). Areas of the artery containing collagen imperfections were discarded as to not interfere with the analysis (see green box in Fig. 4 for an example).

**Figure 5:**
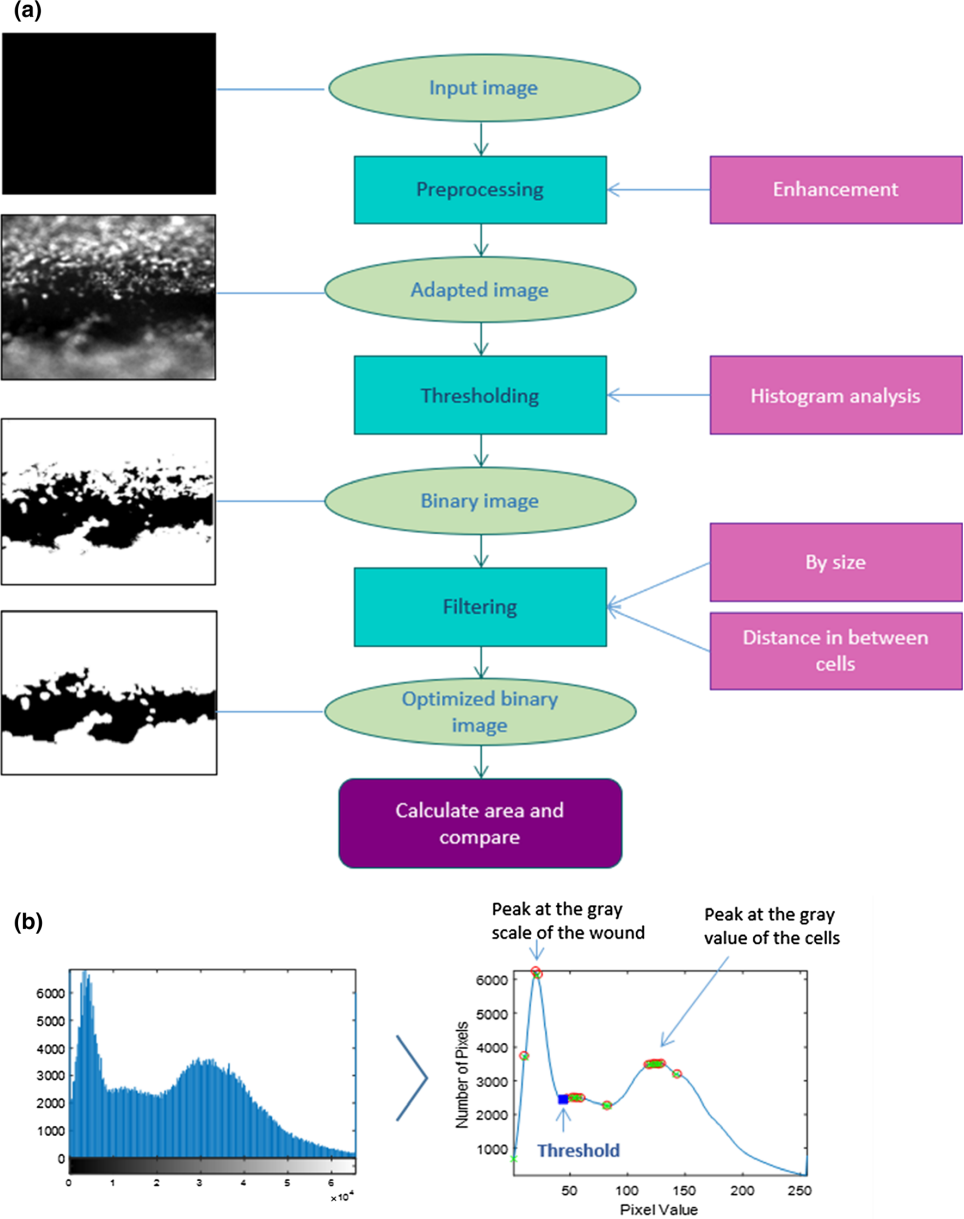
(a) Flow chart illustrating the Matlab-based process used to analyze the wounded area in each ROI. (b) Histogram-based threshold selection.

The wounded area of the artery in each ROI was analyzed independently using a Matlab-based multi-step process as depicted in Fig. 5a. A first step of preprocessing was performed to enhance input images by mapping intensity values, expanding histograms, and improving contrast. Adapted images were processed under a histogram-based segmentation. This technique uses the histogram to select gray levels for grouping pixels into two regions. Two areas were defined in each ROI: the wounded lumen and the endothelium. The wounded area presented low values in the grayscale, while the pixels containing cells contained high grayscale values. Consequently, the histogram presented a high peak at the gray value of the wound and a second smaller peak at the gray value of the cells. For each ROI, the histogram of pixel intensities was first converted from a 16-bit to an 8-bit grayscale and then smoothed with a moving-average filter (window size 10), computing averages of the data contained in each window as depicted in Fig. 5b. To segment the image and separate both areas, the gray value corresponding to the valley after the first peak in the histogram (blue dot in Fig. 5b) was chosen as the threshold and the image binarized. This method proved highly robust and effective in delineating wounded and healed areas.

Further optimization was performed by filtering small elements by size, including isolated cells in the wounded area and small gaps between cells. This optimization involved the following step-by-step process: beginning with the binary image, a filter to remove small objects in the wounded area corresponding to noise or isolated cells was first applied in Matlab using the function bwareafilt. A second filter step was then applied to the previous image to remove small objects in the endothelium. A final step involved removing small objects (gaps) surrounded by cells very close to the front line of the wound. This was accomplished by calculating the Euclidean transform of the previous binary image and filtering out all objects whose dimensions were smaller than half of a typical cell size.

The wounded area was computed from the optimized binary image, allowing quantification of the size of the wound over time for each ROI. Finally, the 4 ROIs in the upstream regions and the 8 ROIs in the stented area were averaged to reduce noise and single events.

### Demonstration of the Validity of the Automated Image Analysis Algorithm

**Figure 6:**
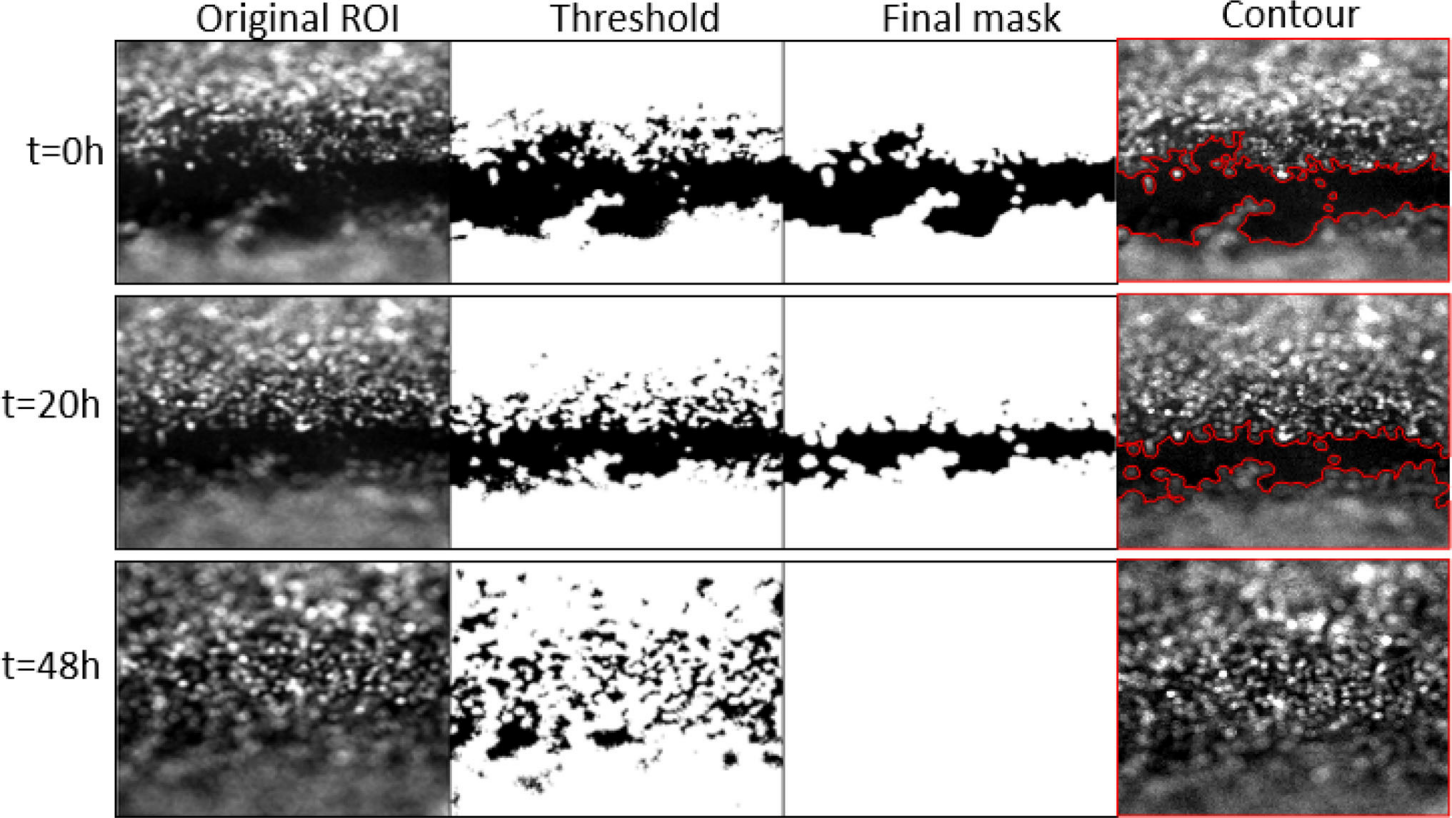
Evolution of the wound and the mask from which the wounded area is calculated over time. Images follow an enhancement process (Original ROI) prior to histogram analysis to obtain the threshold value. Images are divided by this threshold value (Threshold). A final step of filtration produces the final image (Final mask) from which the area covered (and not covered) by cells is calculated to obtain the wound healing rates. The fourth column (Contour) shows the contour (in red) of the final mask overlayed onto the Original ROI. Comparison of the final mask to the original ROI demonstrates the capability of the Matlab routine to accurately capture the wounded area on the original ROI.

The accuracy of the method was investigated by comparing the wounded area obtained with the Matlab routine to that obtained by manual interactive selection of the wound in Fiji for several ROIs. Several time points were selected to perform the comparison as shown in Fig. 6. The results of this comparison indicated that the automated Matlab routine is capable of accurately capturing the wounded area as depicted by the overlay of the computed contours (in red) over the original ROIs.

### Head-to-Head Comparison of Endothelial Wound Healing in the Two Different Stent Types

**Figure 7:**
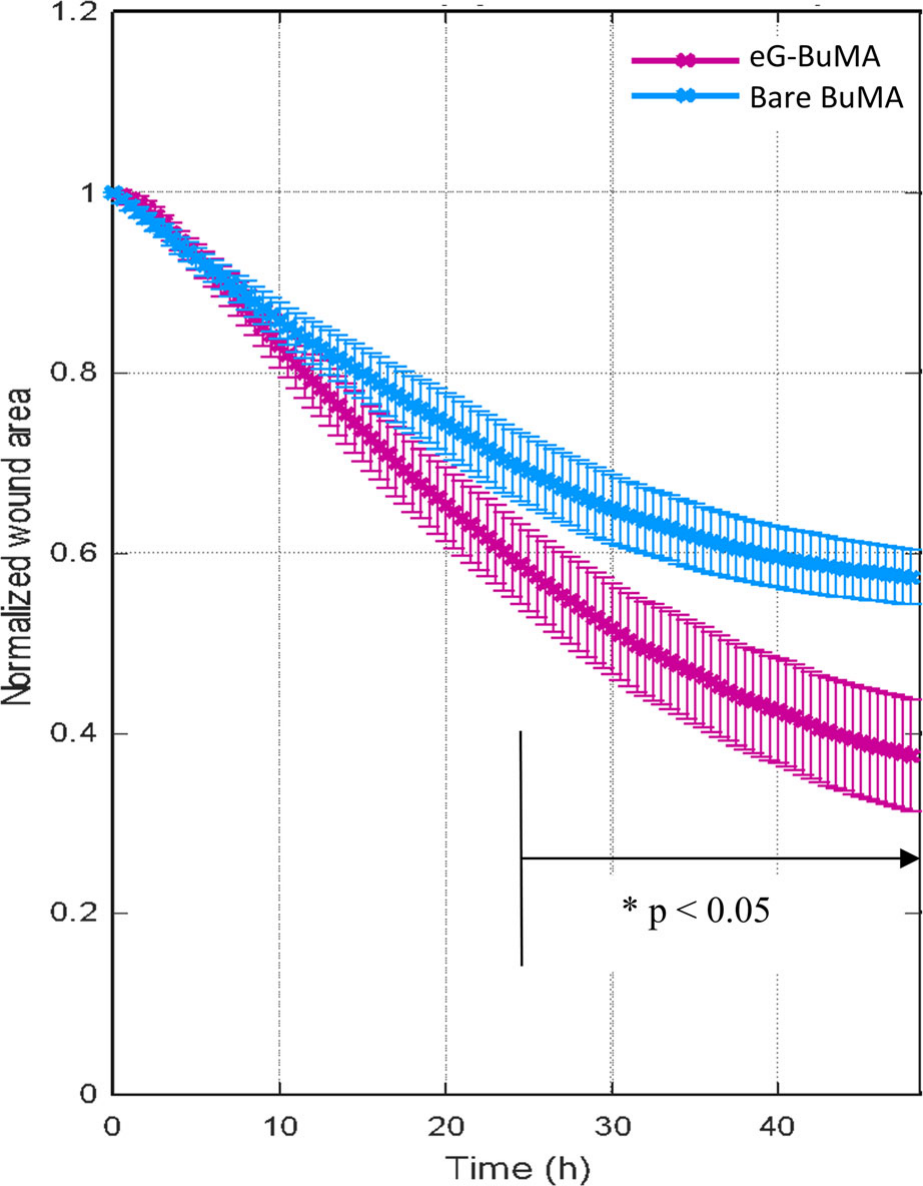
Mean values of normalized wound area for each stent type over time. Bare metal stents (in blue) provided a lower healing rate in comparison with the BuMA eG-BMS stents (in purple). Data are based on five experiments for each type of stent. *Statistically significant differences (*p* < 0.05) which were observed for all time points after *t* = 24.5 h.

The endothelial wound healing process after deployment of the Bare BuMA and eG BuMA stents can be imaged and quantitatively assessed. An example of the time-lapse images for an eG BuMA stent can be visualized in the supplementary material video S2. Figure 7 depicts the evolution in time of the normalized wound area (normalization is relative to the wound area at the first time point) for each stent type (mean ± SEM) during the 48-h recording period. While the wound healing rates for the two stent types appear virtually indistinguishable during the first 10 h, the curves begin diverging at that point with faster wound healing for the eG BuMA stent. The extent of the divergence between the two curves increases progressively with time. More specifically, at the 20-h time point, the normalized wound area is 0.74 ± 0.03 for the Bare BuMA stent and 0.65 ± 0.04 for the eG BuMA stent. The corresponding values at the 40-h time point are 0.60 ± 0.03 for the Bare BuMA stent and 0.42 ± 0.06 for the eG BuMA stent. At the 48-h time point, they are 0.57 ± 0.03 for the Bare BuMA stent and 0.38 ± 0.06 for the eG BuMA stent. Thus, by the end of the experiment, wound healing rates were 44% faster for the eG BuMA stent than for the Bare BuMA stent. The differences in wound healing rate between the two stent types became statistically significant (*p* < 0.05) at all time points beyond 24.5 h. These results indicate that arteries with the eG BuMA stents have an EC wound healing rate that is faster than the case of the Bare BuMA stent.

## Discussion

In an effort to gain insight into the basis for the seemingly excellent performance of the BuMA Supreme® SES, we investigated if the thin (~ 200 nm-thick) PBMA brush layer that is electro-grafted onto the Co–Cr frame of the BuMA Supreme® SES has an impact on stent strut coverage. To this end, we compared endothelial wound healing rates for Co–Cr stents that have the BuMA Supreme® design either with or without the electro-grafted layer (eG BuMA and Bare BuMA stents, respectively). The comparisons were performed using automated analysis of images of fluorescently labeled ECs in an *in vitro* coronary simulated arterial model that we had previously described within which endovascular stents can be deployed and cellular responses monitored continuously.^3^

The first key result of the present study is that quantitative assessment of endothelial wound healing rates within the simulated arterial model is achievable using the automated Matlab-based image analysis routines developed here. The second key result is that endothelial wound healing during the first few days post stent deployment is significantly more rapid for the eG BuMA stent than for the Bare BuMA stent. Interestingly, a recent report indicated that the BuMA Supreme® SES is associated with faster redevelopment of endothelial barrier function in rabbits than a durable polymer everolimus-eluting stent at the 45- and 90-day time points after stent implantation.^6^ Although these findings appear consistent with our present observations, the precise relationship between these two sets of findings remains to be elucidated. In addition to the PBMA coating, the BuMA Supreme® SES also features a shorter drug release time than other DES. Therefore, it is important to delineate the separate contributions of the limited drug release time and the PBMA coating towards the seemingly improved endothelialization profile of the BuMA Supreme® SES. This is indeed what we tried to accomplish here by studying stents that did not contain the drug layer. Nevertheless, the drug and its release dynamics might also be expected to have a significant effect on endothelial wound healing, and this issue certainly merits future investigation.

It might seem that the effect of the PBMA layer on endothelial wound healing would become apparent only after the drug payload has been fully discharged and the biodegradable polymeric topcoat within which the drug is loaded has fully degraded. However, it should be recognized that similar to other biodegradable polymers (with the exception of poly-ortho esters), PLGA undergoes bulk and not surface degradation. Therefore, holes form inside the PLGA layer very early in the degradation process, leading to a situation where the PBMA coating becomes accessible to the outside medium and to the migrating ECs well before the PLGA layer has fully degraded. As a result, investigating the effect of the PBMA coating on endothelial wound healing is thought to be relevant even at early time points after stent deployment.

The mechanistic basis of the improved endothelial wound healing profile associated with the eG BuMA stent remains unknown at this point. Several cellular processes contribute to wound healing including cellular spreading, migration, and proliferation. In light of the fact that the only difference between the eG BuMA stent and the Bare BuMA stent tested here is the presence of the thin PBMA layer, it would be important to explore the possible impact of PBMA on the cellular processes governing wound healing. It is noteworthy that recent reports have demonstrated that PBMA is a biocompatible substrate that favorably supports the adhesion and proliferation of renal epithelial cells.^13^ Another phenomenon that may have contributed to the observed differences in wound healing between the Bare BuMA and the eG BuMA stents and that merits future study is potential differences in the extent of adsorption of serum proteins to the stent surface. Yet another consideration is possible differences in the release from the stent surface of specific ions (such as Ni^2+^) that have a pro-inflammatory effect. It would be interesting to explore if the PBMA coating acts as a shield against the release of such ions and thus serves a cellular protective purpose. Finally, the PBMA coating is expected to have introduced some nano-scale roughness to the stent surface, which may affect the efficiency of EC migration.

Another determinant of endothelial wound healing rate is the local fluid dynamic environment to which the cells are subjected. For instance, previous studies have suggested that low levels of wall shear stress and disturbed flow patterns retard the healing of ECs after mechanical injury.^16^,^20^ Thus, it would be interesting to establish if the very thin PBMA coating has an impact on the microscopic flow structure in the vicinity of the stent struts and whether such an effect may contribute to the observed improvement in wound healing.

The experiments reported here focused on a period of a few days after stent implantation. It remains unknown if the difference in wound healing rate observed here will persist at longer times. Therefore, longer term experiments are certainly warranted. However, extending the duration of the study to a time period of weeks would be quite challenging in the present system because of the difficulty in maintaining cultured cells healthy and viable over extended periods of time.

It is recognized that the experimental system used here represents a great idealization of the very complex environment within which stents are implanted *in vivo.* The wall of the simulated arterial model consists of a simple and very soft collagen hydrogel that does not contain an atherosclerotic plaque, an environment that is mechanically and biochemically very different from the case of a diseased and oftentimes calcified arterial wall within which a stent would typically be deployed. Furthermore, the fluid used in the present study, cell culture medium, is considerably simpler than blood. While these simplifications represent limitations of our *in vitro* simulated arterial model, they also provide a more controlled environment within which individual parameters are more easily manipulated.

An additional conclusion of the present study is that combining an *in vitro* simulated artery model such as the one described here with automated image analysis provides a robust and powerful platform for direct head-to-head comparison of stent performance. Such a system is viewed as a particularly valuable tool for the development and testing of future stent designs. While the present paper focused exclusively on endothelial wound healing, the comparisons afforded by this system can be readily extended to a host of other endpoints such as stent-induced flow disturbance in the vessel lumen, the extent of stent- and/or balloon-induced arterial wall damage, the biocompatibility of stent materials, and the transport within the arterial wall of drugs eluted by stents and/or balloons.

## Supplementary Information

Below is the link to the electronic supplementary material.

Supplementary material 1 (AVI 10821 kb)

Supplementary material 2 (AVI 2983 kb)
